# *Ganoderma* spp.: A Promising Adjuvant Treatment for Breast Cancer

**DOI:** 10.3390/medicines4010015

**Published:** 2017-03-15

**Authors:** Ivette J. Suárez-Arroyo, Yaliz Loperena-Alvarez, Raysa Rosario-Acevedo, Michelle M. Martínez-Montemayor

**Affiliations:** 1 Department of Biochemistry, Universidad Central del Caribe, School of Medicine, P.O. Box 60327, Bayamón, PR 00960-6032, USA; ivette.suarez@uccaribe.edu; 2 Department of Science, Pontificia Universidad Católica-Mayagüez, P.O. Box 1326, Mayagüez, PR 00681, USA; yaliz_loperena@pucpr.edu; 3 Department of Neuroscience and Regenerative Medicine, Augusta University, 1120 15th St, Augusta, GA 30912, USA; Rrosarioacevedo@augusta.edu

**Keywords:** *Ganoderma*, breast cancer, cell death, invasion, migration, metastasis, chemoresistance, natural medicine, NF-κB, PI3K/AKT/mTOR

## Abstract

For the past several decades, cancer patients in the U.S. have chosen the use of natural products as an alternative or complimentary medicine approach to treat or improve their quality of life via reduction or prevention of the side effects during or after cancer treatment. The genus *Ganoderma* includes about 80 species of mushrooms, of which several have been used for centuries in traditional Asian medicine for their medicinal properties, including anticancer and immunoregulatory effects. Numerous bioactive compounds seem to be responsible for their healing effects. Among the approximately 400 compounds produced by *Ganoderma* spp., triterpenes, peptidoglycans and polysaccharides are the major physiologically-active constituents. *Ganoderma* anticancer effects are attributed to its efficacy in reducing cancer cell survival and growth, as well as by its chemosensitizing role. In vitro and in vivo studies have been conducted in various cancer cells and animal models; however, in this review, we focus on *Ganoderma*’s efficacy on breast cancers. Evidence shows that some species of *Ganoderma* have great potential as a natural therapeutic for breast cancer. Nevertheless, further studies are needed to investigate their potential in the clinical setting and to translate our basic scientific findings into therapeutic interventions for cancer patients.

## 1. Introduction

Research proposes that different *Ganoderma* species (*Ganoderma* spp.), including *G. lucidum*, *G. sinense*, *G. atrum*, *G. tsugae*, *G. neo-japonicum* and, most recently, *G. hainanense*, carry promising anticancer properties. Bioactive substances isolated, characterized and identified from *Ganoderma* spp. include triterpenoids, polysaccharides, nucleosides, sterols, proteins and alkaloids. However, two main active ingredients, triterpenes and polysaccharides, have been shown to have significant anticancer effects in vitro and in vivo.

Triterpenes are compounds composed of one or more isoprene units and having anti-inflammatory and antitumorigenic activity [1]. *G. lucidum* possesses over 140 species of triterpenes and triterpenoids [1,2]. Triterpenes are originally isolated from *Ganoderma* spp. spores, and studies demonstrate outstanding therapeutic and pharmacological activities on various diseases, including cancer [3,4]. Studies confirm that subtypes of triterpenes extracted from *G. lucidum* may affect the viability of human cancer cell lines [4]. 

*Ganoderma* contains a significant number of polysaccharides (i.e., β-d-glucans) and peptidoglycans. Polysaccharides extracted from fruiting body, mycelia and spores usually consist of arabinose, galactose, glucose, xylose and mannose [5]. Studies demonstrate that *Ganoderma* polysaccharides exert anticancer effects in tumor therapy by enhancing the immune system [5]. Antitumor and antiglycemic effects, immunomodulation, antiviral activity, protective effects from free radicals and the reduction of cell damage by radiation are attributed to triterpenes and polysaccharides.

Breast cancer (BC) is the leading cause of cancer death in women in the U.S. Cancer is a disease distinguished by clinical behaviors, risk factors, molecular subtypes and response to treatment. BC molecular subtypes identified via gene expression profiles have provided information that has led to the biomarker identification that may facilitate prognosis and treatment [6,7,8]. For example, the presence or absence of estrogen receptor (ER), progesterone receptors (PR) and human epidermal growth factor receptor 2 (HER2) serve as molecular and pathological markers for treatment [8]. About 40% of BC are luminal A, thus making this the most common type of breast cancer [9]. These tumors are less aggressive and tend to have hormonal receptors present, thus having a favorable response to therapy [10]. About 10%–20% are luminal B and tend to be highly proliferative tumors [9,10,11,12]. Basal-like breast tumors account for ~10%–20% and are common in women positive for BRCA1 gene mutation, as well as African American and premenopausal women [9]. Because most of the basal-like cancers tend to be triple negative (no hormonal or HER2 receptor), their prognosis tends to be grim since for these tumor subtypes, no targeted therapies exist. Finally, ~10% of BC express HER2, while not expressing hormone receptors [9]. Similar to basal-like cancers, these tumors grow more aggressively and are associated with poorer prognosis [10]. Importantly, the availability of targeted anti-HER2 therapies has somewhat reversed the adverse prognosis.

Conventional therapy targeted to commonly deregulated signaling pathways in BC is a highly effective remedial strategy. Nevertheless, its usefulness is somewhat limited by intrinsic and acquired mechanisms of resistance, as well as by the fact that BC survivors who have received therapeutic interventions may develop conditions that affect their quality of life (QOL) and their overall survival (OS). Alterations in cell cycle and cell survival pathways and the evasion of apoptotic processes provide tumors with alternative proliferative and growth stimuli. Among pathways associated with therapy resistance are the phosphoinositide 3-kinase/AKT/mammalian target of rapamycin (PI3K/AKT/mTOR), mitogen-activated protein kinases (MAPK), the epidermal growth factor receptor family (ErbB1, also known as EGFR; ErbB2, also known as HER2; ErbB3; and ErbB4) and nuclear factor kappa B (NF-κB). Therefore, research has led investigators to assess the efficacy of alternative methods that sensitize resistant cells to therapy and that enhance QOL. *Ganoderma* spp. has been used in multiple in vitro and in vivo models because of its antiproliferative and growth inhibitory efficacy. In this review, we discuss the use of various *Ganoderma* spp. in BC models, patients and survivors to show the potential this medicinal mushroom has for its use in the clinical setting as a therapeutic for this deadly disease.

## 2. In Vitro Studies

### 2.1. Cytotoxic, Antiproliferative, Cytostatic and Antiapoptotic Effects of Ganoderma spp.

Cancer is characterized by a cascade of critical events that end in uncontrolled cell expansion and invasion. Deregulated cell proliferation together with the obliged compensatory suppression of apoptosis are necessary to support further tumor progression. Most of the chemotherapeutics are cytotoxic or cytostatic, causing toxicity or stopping the cancer cells from multiplying. Cell division is time regulated by the cyclin-dependent kinases (CDKs). Once the cyclins (the CDKs’ regulatory subunits) are bound to the CDKs, they form heterodimeric complexes. Cyclins may also be bound by CDK inhibitors that are responsible for cell cycle deceleration. The cell cycle progresses from the G1 to synthesis (S) to G2 to the mitosis (M) phase, while cells in G0 remain in a non-dividing state [13]. Cell proliferation and death are processes that are regulated to maintain homeostasis. Manipulation of the cell cycle may result in apoptosis prevention or induction; an association has been recognized for tumor suppressor genes, such as p53 and retinoblastoma (RB), c-Myc and various CDKs [14]. Apoptosis is a programmed cell-death process that occurs in normal development and turnover. It is characterized by the activation of endonucleases following the cleavage of chromatin DNA, followed by nuclear shrinkage, condensation of chromatin, membrane blebbing and DNA fragmentation [14,15]. However, improper regulation of apoptosis occurs in a variety of pathological disorders, such as cancer [14].

Whole mushroom extract or individual bioactive compounds of *Ganoderma* have been associated with cell death induction or cell cycle arrest of several human BC cells. In a study where aqueous extracts of *G. lucidum*, *G. sinense* and *G. tsugae* were used to assess their effectiveness against BC cells, data showed significant antiproliferative activities in MCF-7 cells and MDA-MB-231 in a concentration-dependent manner. Among the species tested, *G. tsugae* extract was most potent against MCF-7 cells, whereas the potencies for MDA-MB-231 cell proliferation inhibition were similar among the *Ganoderma* species tested. The extracts did not cause any cytotoxic effect on human noncancerous mammary epithelial (HMEC) cells [16]. Because of the accessibility of the commercially available products of *Ganoderma*, they have also been evaluated to determine their effectiveness against BC. *G. lucidum* in the form of powdered extract (20:1) with spores, which contains 13.5% polysaccharides and 6% triterpenes (ReishiMax GLp^®^, GLE), or the spore-only powder suppressed the proliferation of MDA-MB-231 cells in a dose- and time-dependent manner [17,18]. Treatment with GLE induced cell-cycle arrest at the G0/G1 phase, which was the result of the downregulation of cyclin D1 and CDK4 [17]. GLE also demonstrated a potent cytotoxic effect against noninvasive, estrogen-dependent MCF-7 than the highly invasive, estrogen-independent MDA-MB-231 BC cells [19]. GLE also showed strong cytotoxic effects in the triple negative BC cell lines, SUM-102 and MDA-MB-468, and also on the HER2 overexpressing MDA-MB-435. However, the noncancerous mammary epithelial cells, MCF-10A, were not affected by treatment [20,21]. Furthermore, the effect of GLE has been evaluated in inflammatory breast cancer (IBC), which is the most lethal type of advanced BC and which presents unique characteristics that differentiate it from non-IBCs [22]. In these studies, GLE significantly affected the viability or proliferation of SUM-149 and KPL-4 IBC cells by proapoptotic effects [20,21]. The authors demonstrated that the regulatory gene expression of cell-cycle progression was affected in SUM-149 IBC cells. Treatment with GLE decreased the expression of (cyclin D1) CCND1 and WEE, while it significantly decreased the abundance of (cyclin A2; B2) CCNA2 and CCNB2 cell cycle gene expression [23]. Gurunathan et al. showed that biologically-synthesized silver nanoparticles using *G. neo-japonicum* Imazeki mycelia aqueous extracts induced cell death through the generation of reactive oxygen species (ROS), caspase 3 activation and DNA fragmentation in MDA-MB-231 BC cells [24]. BreastDefend™ (BD) is a dietary supplement composed of various botanicals, including medicinal mushroom extracts (*Coriolus versicolor*, *G. lucidum*, *Phellinus linteus*), medicinal herbs (*Scutellaria barbata*, *Astragalus membranaceus*, *Curcuma longa*) and purified biologically-active nutritional compounds (diindolylmethane and quercetin). Studies show that BD suppressed proliferation of BC cells (MDA-MB-231) in a dose- and time-dependent manner predominantly through cytostatic effects. In an effort to elucidate which genes are responsible for BD’s cytostatic effect, a cDNA microarray analysis validated that BD increased the expression of GADD45A, which affects cell cycle growth arrest and downregulates the expression of cyclin A1 [25].

Alcohol extracts from *Ganoderma* spp. have been evaluated in BC, showing inhibitory results. Moreover, a dose- and time-dependent inhibition mediated through p21/Waf1 upregulation and cyclin D1 downregulation was obtained with a *G. lucidum* ethanolic extract. In this study, the extract significantly increased the expression of Bax (proapoptotic protein), while there was no change in the expression of Bcl-2 (antiapoptotic protein). The researchers demonstrated caspase-7 cleavage along with cleaved PARP expression after 36 h of treatment [26]. Proliferation assays in four tumor-cell lines, including MCF-7 and MDA-MB-231 BC cells, evaluated the effects of the ethanol extracts of *G. lucidum* and *G. sinense*. Human normal fibroblastic cells, Hs68, were used as the control. The study revealed that the *G. lucidum* antiproliferative effect was stronger than *G. sinense* extract and was more effective on the MDA-MB-231 BC cells. Moreover, *G. lucidum* significantly decreased G1/S phase transition, while *G. sinense* induced a cell cycle arrest at G2. As determined by terminal deoxynucleotidyl transferase dUTP nick and labeling (TUNEL) assay, both extracts induced apoptosis even at the lower dose of 40 µg/mL. Triterpenoids, sterols and nucleosides may contribute to the apoptotic induction [27]. The effect of alcohol extract of *G. tsugae* (GTE) was assessed in HER2-overexpressing cancer cells. The study evidenced the antiproliferative effects of the extract on BT-474 and SKBR-3 BC cell lines. Antiproliferative effects in SKBR-3 cells resulted in an increase in G1 with a decrease in S and G2/M phases via regulation of cyclins D1 and E [28]. Wu et al. assessed the anticancer effects of water or ethanol extracts of five different fungal species, including *G. sinense*. The results showed that water extracts exert moderate anticancer activities compared to that of ethanol extract in MDA-MB-231 cells [29]. Recently, a group of researchers from Turkey examined the cytotoxic effects of *G. lucidum* extracts obtained with five different solvents (ethanol-water, methanol, ethanol, ethyl acetate and ether) on MCF-7 cells at 24, 48 and 72 h. Based on the cytotoxicity results, they determined that *G. lucidum* ether extract (G.Ether) had greater potency (IC_50_ = 100 μg/mL at 72 h) against BC cells than the others [30,31]. To study the mechanism behind the extracts’ anticancer effects, they did telomerase activity assays and found a 32% decrease in telomerase activity in treated cells [31].

Many scientists have attributed the anticancer effects of *Ganoderma* spp. to the triterpenes. Ganodermanontriol (GDNT) is a biologically-active triterpene alcohol isolated from *G. lucidum*. To evaluate the antiproliferative effects of GDNT, MDA-MB-231 cells were treated with increasing concentrations of the triterpene for 24, 48 and 72 h, and proliferation was determined. GDNT significantly inhibited the proliferation of cells with an IC_50_ of 42.0, 15.7 and 11.6 µM at 24, 48 and 72 h, respectively, accompanied by a reduction in colony formation. In addition, GDNT suppressed MCF-7 cell proliferation while slightly affecting the proliferation of MCF-10A noncancerous mammary epithelial cells [32]. Two related complexes closely related to E3 ubiquitin ligase, the anaphase-promoting complex (APC) and the Skp1-Cullin1-F-box complex (SCF), are major driving forces controlling cell cycle progression. APC, the most complex E3 ubiquitin ligase, consists of at least 14 subunits, one of them being the coactivator, CDC20 (cell division cycle 20 homologue), which has been associated with oncogenesis [33]. Jiang et al. found the overexpression of CDC20 in BC cells and tissues. Interestingly, GDNT treatment resulted in suppressed CDC20 expression in MDA-MB-231 BC cells [32]. The anti-BC activity of the ethanol-soluble and acidic component (ESAC), which mainly contains triterpenes extracted from *G. lucidum*, was determined after 48 h of treatment on MCF-7 and MDA-MB-231 cells. The results showed that ESAC notably decreased the viability of BC cells in a concentration-dependent manner, mediated by G1 cell cycle arrest and apoptosis, as demonstrated by the condensation of nuclear chromatin, DNA fragmentation and increased expression of a PARP cleavage [34]. Moreover, a study testing ganoderic acid DM (GADM), a *G. lucidum* triterpenoid, showed a dose- and time-dependent decrease in cancer cell viability [35]. Wu et al. showed that a low concentration of the GADM triterpene extract effectively induces a G1 cell cycle arrest in MCF-7 cells [35]. Interestingly, this study revealed that MDA-MB-231 cells need higher concentrations of the triterpenoid extract to mediate G1 cell cycle arrest. This effect may be attributable to the greater proliferative and metastatic potential of these cells. The researchers confirmed their results by assessing the expression of the catalytic subunits of the CDK complex, CDK2 and CDK6, which are essential for the G1/S transition, as well as the expression of cyclin D1 and retinoblastoma (Rb) phosphorylation. GADM downregulated the abundance and p-Rb in a time-dependent manner [35]. Similarly, ganoderic acid ME (GA-Me) purified from *G. lucidum* inhibits cell proliferation and induces apoptosis of BC cells by decreasing the prosurvival proteins BCL-2 and c-Myc, as well as the cell-cycle regulator cyclin D1 [36]. The effects on BC cell viability and morphology of *G. lucidum* extract mainly containing ganoderiol A (GA), dihydrogenated GA, and GA isomer (GAEE) was studied in highly metastatic BC cells. GAEE exhibited no toxicity in MDA-MB-231 BC cells at concentrations between 5 µg/mL and 20 µg/mL. Moreover, GAEE did not induce cell cycle arrest or apoptosis [37]. Recently, Peng et al. isolated 19 lanostane triterpenoids (15 unknown and 5 known compounds) from *G. hainanense*, a rare species of *Ganoderma*. The group evaluated the cytotoxicities of 16 compounds, including ganoderone A, lucidadiol, ganodermanontriol and 4,4,14a-trimethyl-3,7-dioxo-5a-chol-8-en-24-oic acid. Compounds 10, 12 and 17 (lucidadiol) showed inhibitory activities against MCF-7 cells [38]. Polysaccharides do not have direct cytotoxic activity against tumor cells, but do stimulate the immune response. Shang et al. purified a *G. lucidum* polysaccharide extracted from the selenium (Se)-enriched mycelia (SeGLP-2B-1) of the mushroom [39]. The cytotoxic effect of SeGLP-2B-1 was assessed in BC using increasing concentrations of polysaccharides for 24–72 h. The results showed that at 24 h, MCF-7 BC cell viability was not significantly decreased. MCF-7 BC cell viability was significantly reduced in a dose-dependent manner mediated by apoptosis after 48 h of treatment. The mechanisms behind this process included the intrinsic and extrinsic apoptotic pathways. Apoptosis results showed the formation of sub-G1 apoptotic bodies mediated by an increase of caspases-8, -9 and -3 and cleavage of PARP. Moreover, the loss of mitochondrial action potential accompanied by the release of cytochrome c into the cytosol paralleled the apoptosis data, suggesting that SeGLP-2B-1 induced mitochondria-mediated cell death [40]. Proteins of *Ganoderma* spp. are also identified as bioactive compounds with immunomodulatory and anticancer activities. One of the most important immunomodulatory proteins isolated from *G. lucidum* is Lhing-Zhi-8 (LZ-8) [41]. LZ-8 is a potent mitogen of T cells and peripheral blood mononuclear cells (PBMCs), an activator of macrophages and of human monocyte-derived dendritic cells and an inducer of cytokines [42,43,44,45]. LZ-8 has also immunosuppressive effects in vivo, showing a reduction in antibody production [46]. The antitumor effects of LZ-8 were also demonstrated in human lung cancer. Results from this study showed that recombinant LZ-8 (rLZ-8) reduced the proliferation of A549 human lung cancer cells, inducing a G1 cell-cycle arrest mediated by the overexpression of p53 and p21. Moreover, rLZ-8 decreased tumor growth in xenograft models of Lewis lung carcinoma [47]. In addition, the anticancer effects of LZ-8 were evidenced in human gastric cancer cells. rLZ-8 induced SGC-7901 gastric cancer cell death by autophagy mediated by the activation of the ER-associated degradation systems (ERAD) in a caspase-independent manner [48]. An active fucose-containing glycoprotein fraction isolated from a water-soluble Ling-Zhi (*G. lucidum*) extract (FFLZ), exerts immunomodulating activities by stimulating the expression of inflammatory cytokines and antibody-mediated cytotoxicity in cancer cells [49,50]. Tsao et al. evaluated the effects of FLZZ on the growth of mouse BC cells 4T1 and on MDA-MB-231 and showed that treatment decreased the viability of both cell lines in a dose-dependent fashion. Furthermore, FFLZ-treated 4T1 cells formed fewer colonies than untreated cells, indicating that FFLZ inhibits the colony formation of 4T1 cells [51]. Khz is a crude polysaccharide isolated from the fusion of *G. lucidum* and *Polyporus umbellatus* mycelia. This protein selectively induced apoptosis in cancer cells by increasing intracellular [Ca^+2^] to generate ROS [52]. Recently, the same group of investigators showed that Khz decreased proliferation and induced apoptosis on MCF-7 cells following similar mechanisms evidenced before. The results also demonstrated that Khz proapoptotic effects were mediated by caspase-7, -8 and -9 [53]. Fungal immunomodulatory proteins (FIPs), which are small-molecule proteins isolated from higher basidiomycetes, have a variety of biological functions, including hemagglutination, antianaphylaxis, antitumor and immune-regulating activity. Currently, eight FIPs have been isolated from *Ganoderma* species, such as *G. lucidum*, *G. tsugae*, *G. japonicum*, *G. microsporum*, *G. sinensis* and *G. atrum* [54,55]. A FIP isolated from *G. atrum* (FIP-gat), is a new member of the FIP family. Recently, the anticancer effect of recombinant FIP-gat was evaluated in BC. Results showed that rFIP-gat reduced cell viability of MDA-MB-231 cells in a dose-dependent manner after 48 h and has agglutinating activity. rFIP-gat induced cell cycle arrest in G1 accompanied by proapoptosis effects. To study the effects of rFIP-gat in gene expression, investigators performed a gene microarray and found 669 differentially-expressed genes with a minimum fold change of two upon treatment. They validated 10 genes that were increased by the protein and that play an important role in cell death and cell growth, such as TNFSF8, SQSTM1 and DUSP1 [55]. These data suggest that *Ganoderma* spp. and their bioactive compounds are capable of inducing cytotoxicity, antiproliferative effects, proapoptotic processes and cell cycle arrest (Table 1) as part of its anti-BC-promoting properties.

### 2.2. Antimigration and Anti-Invasion Potential of Ganoderma spp.

A cancer trait is the unique capacity of cells to evolve from a hyperplastic state to an increasingly disorganized and invasive tumor that can eventually propagate to distant organs, thus metastasizing. Additional traits include motile and invasive properties, which are the first steps that a malignant cell utilizes to overcome the noncancerous tissue barriers [56]. Noncancerous tissue requires proper adhesion to the basement membrane or neighboring cells and a signaling network to create a homeostatic environment that is maintained. Cancer cells overcome these barriers via deregulation of the signaling network, resulting in modulation in the expression of proteins involved in basement membrane formation, motility and cytoskeletal remodeling. Cancer cells may detach from the primary tumor either as individual cells or collectively as cell sheets, strands and clusters to travel through surrounding extracellular stroma and to gain entry into blood and lymphatic vessels [56]. 

Anti-invasive properties of *G. lucidum* extracts have been shown in various cancer cell lines, including BC. Contrary to the antiproliferative and chemoresistance roles for which many *Ganoderma* spp. have been tested, anti-invasive or migratory properties in BC models have been studied mainly with *G. lucidum* and are described herein. GAEE was used to examine the anti-invasive effects in the human invasive BC cells MDA-MB-231. Wound-healing assays showed that increasing concentrations of GAEE extract inhibited cell migration as much as 73% when used in concentrations of 20 µg/mL. This effect was attributed to the ability of GAEE to attenuate the binding affinity between focal adhesion kinase (FAK) and the cytoskeletal protein paxillin, which might affect cell migration and adhesion. Moreover, GAEE downregulated RhoA, Rac1 and Cdc42 expression and decreased the interaction between N-WASP and Cdc42 [37]. Thyagarajan et al. evaluated a green tea extract (GTE) containing 97% polyphenols and 38% epigallocatechin gallate (EGCG), individually and in combination with GLE in MDA-MB-231 BC cells [57]. Although the sole GLE (0–500 μg/mL) or GTE (0–125 μg/mL) suppressed migration of MDA-MB-231 cells in a dose-response manner, the combination of GLE and GTE synergistically inhibited cell migration. Furthermore, GLE markedly inhibited invasion through the artificial basement membrane Matrigel^^®^^, and the addition of GTE increased invasion inhibition further, demonstrating a synergistic anti-invasive effect, as well [57]. To evaluate the anti-invasive effects of *G. lucidum*, Sliva et al. used different sources of commercially available extracts composed of whole spores, broken spores, ground-fruiting body particles/mushroom powder or GLE. In this study, they found that all extracts except for the ones composed of just broken spores or mushroom powder were effective in decreasing the adhesion, migration and invasion of MDA-MB-231 by more than 50%. Remarkably, GLE inhibited 99% of MDA-MB-231 migration [58], while aqueous extracts of *G. lucidum* spores or fruiting body both inhibited cell motility [59]. A *G. lucidum* dietary supplement also inhibited cell migration (MCF-7 and MDA-MB-231), cell adhesion to fibronectin and vitronectin and invasion of the metastatic cell line MDA-MB-231 [60]. Studies in IBC also support the anti-invasive effect of GLE. Treatment with GLE inhibited migration and invasion of SUM-149 cells after 24 and 72 h [20,21]. Ganoderic acid, GDNT and GA-Me also reduced BC (MDA-MB-231) cell adhesion to the extracellular matrix protein vitronectin, as well as cell migration and cell invasion [32,36,61]. *G. lucidum* also inhibited oxidative stress-induced migration of MCF-7 BC cells [62]. Recently, a mix of medicinal mushrooms and plants was developed for cancer treatment. Studies using the MycoPhyto^®^ Complex, a mix of six varieties of mushroom (*Agaricus blazei*, *Cordyceps sinensis*, *Coriolus versicolor*, *G. lucidum*, *Grifola frondosa* and *Polyporus umbellatus*) mycelia, plus β-1, 3-glucan isolated from the yeast *Saccharomyces cerevisiae* and BD decreased MDA-MB-231 BC cell migration and invasion [25,63]. The effect of the glycoprotein FFLZ was evaluated in vitro using wound closure and migration/invasion assays. Results indicated that FFLZ significantly reduced MDA-MB-231 cell invasion and mobility [51]. The epithelial mesenchymal transition (EMT) process is the differentiation of epithelial cells into motile mesenchymal cells. This switch in cell behavior is mediated by reprogramming gene expression and cell signaling. During EMT, epithelial cells lose their junctions and cell polarity and reorganize their cytoskeleton. These changes increase their migration and invasion capacity [64]. MDA-MB-231 cells incubated with FFLZ displayed a distinct cell-cell adhesion and cluster morphology, formation of protrusions and destruction of actin filaments characteristic of the EMT process. To examine the mechanisms behind the phenomenon of morphology alteration, the investigators assessed the effects of FFLZ on the expression of EMT markers. They found that FFLZ decreases mesenchymal cell markers (e.g., N-cadherin and vimentin) or increases epithelial cell markers (e.g., E-cadherin and γ-catenin). Moreover, FFLZ decreases the TGFβ-induced Smad2/3-Smad4-Snail/Slug-axis pathway and the expression of the EMT-related transcriptional factors in BC cells [51]. There is growing evidence that tumor cell aggregates or spheroids provide a more representative model of tumors in an in vitro setting than what can be achieved with monolayer cultures. Parameters such as stiffness, pH, oxygen, glucose and growth factors are intentionally misaligned in standard 2D cell culture, making it a poor physiological model for tumor studies [65]. Spheroids create cell-cell connections and have decreased proliferation and increased cell survival rates. They may also show tumor dormancy and a hypoxic core, which are features of the tumor microenvironment [66]. These characteristics give rise to a more stratified composition, with the spheroids consisting of a border of proliferating cells, followed by a layer of quiescent cells [67]. Pathologically, many types of BC form tumor emboli as a mechanism of lymphovascular invasion, which are visualized in three-dimensional (3D) culture as tumor spheroids [68]. SUM-149 IBC cells typically form spheroids in culture when utilizing 3D cell matrixes. Studies show that SUM-149 cells invade as tumor spheroids in the control treatment, while GLE treatment disintegrates tumor spheroids by disrupting cell-cell interactions typically formed by invading cells after 48 or 72 h of treatment [20]. All of the gathered data suggest that *Ganoderma* inhibits the migration and invasive behavior of human BC cells in vitro.

## 3. Signaling Studies

The joint interaction between tumor cells and the reactive stroma strongly contributes to the development and progression of cancer. Cells use signaling cascades to amplify and send messages, generally for regulatory processes, such as control of transcription to maintain homeostasis. In cancer cells, many of these signaling cascades are deregulated or reprogrammed to evade apoptosis, thus maintaining a proliferative potential and resisting therapy. Medicinal mushroom supplements have been effective in signaling cascade modulation and cancer cell sensitization for conventional therapies. In this section of the review, we discuss signaling pathways involved in the oncogenesis of BC cells and how *Ganoderma* spp. and their compounds modulate them (Table 2).

### 3.1. HER2 Signaling Pathways

HER2 is a transmembrane tyrosine kinase receptor belonging to the EGFR family (which also includes EGFR, HER3 and HER4). All members of the EGFR family share a common molecular structure: an extracellular ligand-binding domain with an amino terminal, a single transmembrane spanning region and an intracellular cytoplasmic domain with tyrosine kinase activity. Once the receptor-specific ligand binds to the extracellular domain, the receptor adopts a specific conformation, which permits the receptor to form homodimers or heterodimers between the family members. However, HER2 is an orphan receptor that exists constitutively active. The formation of heterodimers or homodimers thereafter activates the intracellular tyrosine kinases and triggers the autophosphorylation of specific tyrosine residues, ending in the activation of signaling cascades, such as PI3K/Akt/mTOR and MAPK [71]. Although HER2 is vital for normal cell processes, the overexpression of HER2 leads to tumorigenesis. HER2 gene amplification and HER2 protein overexpression accounts for about 25%–30% of all BC and is associated with aggressive behavior, chemotherapy resistance, poor prognosis, a low OS rate and metastasis [72]. Kuo et al. demonstrated that GTE inhibited p-HER2 and p-Akt in SKBR-3, BT-474 and MCF-7/HER2 HER2-BC overexpressing cells. To investigate the mechanisms that underline the GTE-mediated downregulation of HER2, the authors assessed the effects of GTE on HER2 mRNA expression and HER2 protein stability. They found that GTE decreases the expression of HER2 mRNA and shortened the half-life of HER2 [28]. 

### 3.2. PI3K/AKT/mTOR

PI3K is a key intermediate in cell responses induced by various agonistic signals that result from downstream target activation by proteins and lipids [73]. PI3K is composed of the catalytic subunit p110 (α, β and δ) and regulatory subunits of 55, 87 or 101 kDa [74]. Lipids at the plasma membrane serve as docking sites for proteins that have pleckstrin homology (PH) domains, such as AKT. AKT is a central serine/threonine kinase involved in survival mechanisms of the cell by inhibiting apoptosis [75]. Activation of AKT leads to the phosphorylation of mTOR, which in turn activates translation by cap-dependent and independent pathways. The serine/threonine protein kinase mTOR is a cell metabolism, growth and survival central regulator. mTOR is activated in response to mitogenic signals, such as hormones, growth factors, nutrients, energy and stress, which leads to cell growth, proliferation and survival [76]. mTOR effectors are the eukaryotic translation initiation factor 4e binding protein (EIF4EBP1 or 4E-BP1) and the ribosomal protein S6 kinase (p70S6K) [77]. mTOR regulates the eIF4F cap-dependent translation initiation complex, which consists of the eIF4E cap-binding protein, eIF4A RNA helicase and the eIF4G scaffold protein. [77]. In IBC tissues and cells, overexpression of the eIF4GI is observed [78]. Overexpression of eIF4G drives greater cap-dependent translation, plus elevated levels of eIF4G enhances internal ribosome entry site (IRES)-dependent translation. The latter is used as an alternative translation initiation mechanism by a subset of mRNAs. In IBC, eIF4G is accountable for the strong homotypic cell interaction that drives tumor emboli formation and promotes IBC cell invasion [78]. Studies demonstrate that GLE significantly modulates mTOR signaling in SUM-149 IBC cells. This is shown by reduced expression of p-mTOR at Ser2481 (a site that promotes intrinsic catalytic activity) [79]. GLE also reduced p70S6K, S6, p-S6 and p-4E-BP1 expression [23]. Moreover, GLE induced a ~50% reduction in protein synthesis in IBC cells, an effect not seen in GLE-treated MCF-10A noncancerous cells [23]. 

Deregulation of PI3K/AKT/mTOR is associated with increased transformation and oncogenesis [80]. Constitutive activation of PI3K causes migration of MDA-MB-231 by means of both catalytic and regulatory subunits of the PI3K complex [73], an effect that GLE reduced by modulation of the NF-κB pathway [58]. Furthermore, Jiang et al. demonstrated that GLE inhibits AKT expression in a time-dependent manner with no inhibition of p-AKT-Thr308, which corresponds to the residue in the PI3-K activation loop by the phosphoinositide-dependent kinase 1 (PDK1). GLE decreased p-AKT-Ser473, which is the residue activated by mTOR. This reduction in turn suppressed NF-κB activity in MDA-MB-231 BC cells [17]. The effect of GLE was also studied on the PI3K/AKT pathway in SUM-149 IBC cells. GLE downregulated the gene expression of *AKT1*, *CCND1*, *EIF4GI*, *MAPK1* and *HRAS*, while the expression of *JUN* and *FOS* was upregulated after 3 h of treatment [23]. Moreover, in vivo studies investigating GLE effects on IBC tumor lysates showed that GLE reduced the expression of the IBC biomarker, E-cadherin, of p120-catenin and of c-Myc. mTOR signaling protein abundance was significantly reduced in tumor lysates from GLE-treated mice, where mTOR, p70S6K and eIF4G expression was reduced [23]. Because loss of mTOR function impacts MAPK activation [81], the authors evaluated GLE’s effect on molecules from this pathway. Data show that GLE reduced RAS and p-ERK1/2 expression without affecting total ERK1/2 in tumor cell lysates [23].

### 3.3. NFκB

Nuclear factor-κB (NF-κB)/Rel is a group of proteins that comprise NF-κB p52/p100, NF-κB1 p50/p105, c-Rel, RelA/p65 and RelB [82]. NF-κB function as transcription factors that control genes regulating multiple processes, including innate and adaptive immunity, inflammation, stress response, B cell development and oncogenesis [83]. In most cells, NF-κB complexes are inactive, residing predominantly in the cytoplasm sequestered by inhibitory IκB proteins [82]. When this signaling pathway is activated (i.e., upon activation of the PI3-Kinase [84], interleukins or the tumor necrosis factor pathways), the IκB kinase (IKK) complex phosphorylates IκB and tags it for proteasomal degradation, liberating NF-κB, which dimerizes and translocates to the nucleus to modulate target gene expression [85]. NF-κB promotes proliferation and invasion and blocks apoptosis in different cancer types, including human BC [82,86] and activated NF-κB is detected in estrogen receptor-negative human BC cells with overexpressed EGFR [86]. Studies by Sliva et al. show that GLE suppresses the motility of BC cells by inhibiting NF-κB [59]. In accordance with the migration results explained in Section 2.2, the same extracts were also effective in the inhibition of the NF-κB pathway, which was directly linked to the invasion inhibition of this BC cell line [58]. Sliva et al. demonstrated that GLE inhibits the activity of NF-κB in BC cells. The researchers investigated the mechanistic basis of the inhibitory effects of *G. lucidum* on MCF-7 (estrogen dependent) and MDA-MB-231 (estrogen independent) proliferation. They found that GLE downregulates ERα expression, inhibits estrogen-inducible ER transactivation and inhibits tumor necrosis factor-alpha (TNFα)-stimulated activation of NF-κB in MCF-7 BC cells. GLE also decreased the estrogen response element (ERE) and NF-κB constitutive activity in MDA-MB-231 BC cells. Moreover, the ER and NF-κB pathway inhibition led to c-Myc downregulation [19]. As established earlier, GA-Me inhibits cell proliferation, induces apoptosis of BC cells by decreasing prosurvival proteins and decreases cell migration and invasion. GA-Me also has anti-BC tumor and antiangiogenesis properties [36]. The same study indicated that GA-Me inhibited NF-κB activity in the absence or presence of TNF-α without affecting the phosphorylation and degradation of its inhibitor (IkB-α). GA-Me downregulated c-Myc, cyclin D1, Bcl-2, MMP-9 VEGF, interleukin (IL)-6 and IL-8 expression, all of which are NF-κB-regulated genes [31]. 

### 3.4. AP-1

Activator protein 1 (AP-1) is a transcription factor composed of a dimeric complex that contains members of the JUN and FOS (c-Fos, Fra-1, Fra-2 and Fos-B) protein families. Members of the FOS family heterodimerize with members of the JUN family, creating complexes that are transcriptionally active [87]. Once dimerized, AP-1 binds to DNA response elements [tissue plasminogen activator (TPA) response elements and cAMP response elements (CREs)] in the promoter and enhancer regions of specific genes [88]. In vitro studies show that FOS-JUN heterodimers create more stable complexes and display stronger DNA-binding activity versus JUN homodimers [87]. Studies in human BC cells show that GLE suppresses the activity of constitutively-active AP-1, followed by urokinase-type plasminogen activator (uPA) and its receptor (uPAR) downregulation [59]. 

### 3.5. Proteases

The urokinase-type plasminogen activator specifically cleaves the Arg-X-Val bond in the zymogen form to activate the enzyme plasmin [89]. Plasmin mediates invasion directly by degrading collagen IV, fibronectin and laminin or indirectly by MMP 2, 3 and 9 and uPA activation. Moreover, uPA also regulates cell adhesion and chemotaxis [89,90] by stimulation of migration. First, it can act directly through proteolytic activity by transforming growth factor-β (TGF-β) activation [91]. An alternative mechanism involves uPA as a nonproteolytic protein, which stimulates cell migration directly through interaction with its receptor (uPAR) [92]. uPA plays a crucial role in tumor metastasis, and its overexpression in BC is a marker of poor prognosis [93,94]. GLE studies show that the expression of uPA and its receptor uPAR is downregulated upon GLE treatment in BC cells [59], while additional studies with the *Ganoderma* spp. triterpene, ganoderic acid, show uPA/uPAR signaling downregulation via AP-1 and NF-kB activity modulation in MDA-MB-231 BC cells [61,69]. Jiang et al. demonstrated that the antitumor and antimetastases effects of the BD dietary supplement were mediated via downregulation of the plasminogen activator urokinase (*PLAU*) and chemokine (C-X-C Motif) receptor 4 (*CXCR4*) gene expression [70]. 

Another group of proteases important in oncogenesis are the MMPs. These are zinc-dependent endopeptidases that degrade the extracellular matrix. MMPs are key mediators of invasion and metastasis and are involved in cell proliferation, survival, angiogenesis and migration [95]. The MMPs regulate various physiological and signaling events and play a key role in tumor and stroma communication [96]. Studies have evaluated the diagnostic and prognostic value of circulating MMP2 and MMP9 in BC patients because elevated levels of both metalloproteinases have been observed in BC patients’ blood. MMP levels are also correlated with stage and lymph node metastasis [97,98,99,100]. Studies demonstrated that GLE and GA-Me downregulates MMP9 gene expression in SUM-149 and MDA-MB-231 cells [20,36]. Moreover, gelatinase activity in response to 48 h of GLE treatment shows that the activity of MMP2 and MMP9 was significantly inhibited by almost 50% after normalizing for total cell number [20].

## 4. Synergistic Effects between *Ganoderma* spp. and Antineoplastic Drugs

Cancer patients frequently relapse after chemotherapy because of the acquisition of resistance to antitumor drugs. Resistance to cancer cytotoxic agents may occur by several mechanisms, such as poor absorption, inactivating metabolism, reduced drug availability or defective immune-system-mediated functions [101]. As part of the search for agents that could overcome this chemoresistant property, *Ganoderma* spp. plus conventional cancer therapies have been evaluated. Additive and synergistic effects between *Ganoderma* spp. and antineoplastic drugs have been shown in different cancer cells and tumors, such as in Lewis lung carcinoma [102], urothelial carcinoma [103], non-small [104] and small-cell lung cancer [105], chronic myelogenous leukemia [106], colon cancer [107], hepatocellular carcinoma [108], ovarian cancer [28,109], epidermoid carcinoma [110] and sarcoma [111]. 

Drug resistance is also characteristically found in advanced-stage BC. The inability of some BC patients to respond to targeted therapies starting at the beginning of treatment is called de novo drug resistance. In contrast, significant numbers of BC patients initially respond to chemotherapy and then turn refractory in a process of acquired resistance, resulting in poor prognosis [112]. The antitumor effects of bioactive *Ganoderma* spp. compounds present in a variety of BC models, as reviewed herein, suggest its use together with chemotherapeutic agents to overcome the chemoresistant phenotype. In studies using the methanolic extract of *G. tsugae*, the growth-inhibitory effect of taxol and cisplatin in the HER2+ BC cell line MBA-MD-435 was enhanced with 250 µg/mL of the extract [28]. Another study shows that the biologically-active compound ergosterol peroxide, isolated from *G. lucidum*, could overcome drug resistance conferred by miR-378 in MDA-MB-231 cells [113]. In vivo studies using MM46 mammary carcinoma C3H/HeN mice fed with a control or an AIN-93M diet containing 2.5% *G. lucidum* antler form extract and injected with 50 or 150 mg/kg of cyclophosphamide show that combining the cyclophosphamide plus 2.5% *G. lucidum* significantly inhibits tumor growth. Moreover, the respective final tumor weight was decreased to about 50% compared to tumors from mice fed control diets [114]. Recently, the interaction between *G. lucidum* and tamoxifen or doxorubicin was examined in MCF-7 cells, showing a synergistic interaction between the G.Ether and tamoxifen. Interestingly, G.Ether decreased the cytotoxic effects of doxorubicin in ER+ BC cells, showing an antagonistic effect between therapies [30]. Because of the high incidence of tyrosine kinase inhibitors (TKIs) targeted against EGFR resistance, a new study investigated the therapeutic potential of GLE in combination with the EGFR TKI Erlotinib in vitro and in vivo. In these studies, GLE synergizes with erlotinib to sensitize SUM-149 IBC cells to the conventional therapy. Moreover, GLE overcomes intrinsic (MDA-MB-231 BC cells) and developed (rSUM-149 IBC cells) erlotinib resistance. Furthermore, erlotinib/GLE decreased SUM-149 IBC cell viability, proliferation, migration and invasion. GLE increased erlotinib sensitivity via EGFR, AKT and ERK signaling inactivation [21]. These studies evidence that a combinatorial therapeutic approach using *Ganoderma* spp. and traditional therapies may be the best way to increase prognosis in BC patients.

## 5. *Ganoderma* spp. and DNA Damage Protection

In human cells, intrinsic and extrinsic factors, such as UV light, X-radiation, gamma irradiation and ionization, can cause DNA damage. Unrepaired or misrepaired DNA damage can result in the accumulation of genetic insults, leading to neoplastic transformation. Several studies have shown the radioprotective properties of *Ganoderma* spp. Studies have shown that using polysaccharides isolated from *G. lucidum* enhanced the repair process after gamma irradiation treatment using in vitro and in vivo models [115,116]. Lanostanoids isolated from *G. tsugae* protected human cells against damage induced by UVB light [117]. Moreover, a water-soluble extract of *G. lucidum* increased small intestinal crypt survival and enhanced body weight and complete blood counts of irradiated mice from radiation damage after X-irradiation [118,119]. 

### Antioxidant Potential of Ganoderma spp.

The production of reactive compounds is the result of the oxidative metabolism in aerobic respiration. These normally low concentrations are necessary for enzyme activation, gene expression, disulfide bond formation during the folding of new proteins in the endoplasmic reticulum, signal transduction and caspase activity control. However, <5% of them may cause cell toxicity if their concentration increases by internal or external sources [120]. Reactive species can be classified into four groups based on the main atom involved. ROS are the most abundantly produced. When the pro-/anti-oxidant equilibrium is lost, oxidative stress occurs, which alters and damages DNA, RNA, lipids and proteins. ROS cause malfunctions in DNA repair and mutations in the DNA, enhancing aging and carcinogenesis. Antioxidants scavenge reactive species through the enzymatic superoxide dismutase (SOD), catalase (CAT) and glutathione peroxidases (GPXs) [120]. Deepalakshmi et al. evaluated the in vitro and in vivo antioxidant potential of *G. lucidum* fruiting-body ethanolic extract on 7,12-dimethylbenz(a)anthracene (DMBA)-induced mammary carcinogenesis in Sprague–Dawley rats. In vitro antioxidant and radical scavenging assays show that the extract had good scavenging activity. In vivo antioxidant enzymatic levels of SOD, CAT and GPx decreased in DMBA-induced animals. Furthermore, extract pretreatment in DMBA-induced animals significantly increased SOD, CAT and GPx levels in plasma, mammary and liver tissues [121]. These data evidence the potential of *G. lucidum* to be a natural source of antioxidants and a chemopreventive agent against BC.

## 6. In vivo Studies

### 6.1. Antitumor and Antimetastasis Effects of Ganoderma spp.

The invasion-to-metastasis transition is a multistep process that consists of several cell-biological changes. This transition starts by local invasion, followed by cancer cell intravasation, transit through the lymphatic and circulatory systems, extravasation, the formation of small colonies of cancer cells at the distant site (micrometastases) and colonization of the new organ [122]. 

We have already discussed that BD inhibits MDA-MB-231 cell proliferation, migration and invasion. However, to evaluate whether BD suppresses tumor growth and breast-to-lung metastasis, the authors used a human BC orthotopic model. Female immunocompromised mice were injected with 1 × 10^6^ MDA-MB-231 cells into the mammary fat pad. After 1–2 weeks of implantation, mice were orally gavaged with 0, 100, 200 and 400 mg BD per kg/BW for 33 d. After treatment, mice treated with the highest concentration of BD demonstrated a decrease in body weight in comparison with the control group. However, the necropsy did not show signs of toxicity, and liver, spleen, kidney, lung and heart weights were not different between the treatments. Furthermore, no abnormalities were seen in those organs. The lowest concentration of BD (100 mg per kg/BW) significantly decreased the tumor volume over time and the incidence of breast-to-lung cancer metastasis by 70% [70]. Because the natural supplement ReishiMax GLp^®^ selectively decreases viability, migration and invasion of IBC cells, a study of the antitumor effects of GLE in a SUM-149 severe combined immunodeficient (SCID) xenograft model was performed. After 13 weeks of 28 mg/kg BW GLE via oral gavage, GLE-treated mice showed a 45% reduction in tumor weight and a 50% reduction in tumor volume when compared with vehicle-treated mice [23]. The antitumor activity of the extract was assessed via tumor lysate PCR arrays, and results showed eukaryotic initiation factor 4B and ribosomal protein S6 kinase, 70 kDa, polypeptide 1 (*EIF4B*, *RPS6KB1*), gap junction protein alpha 1, 43 kDa (*GJA1*), p21 protein (cdc42/Rac)-activated kinase 1 (*PAK1*) and pyruvate dehydrogenase kinase, isozyme 1 (*PDK1*) expression downregulation. Also in that study, the nuclear factor of kappa light polypeptide gene enhancer in B-cells inhibitor, alpha (*NFKBIA*) gene was upregulated [23]. Li et al. evaluated the antitumorigenic effect of GA-Me using xenograft models of BC. SCID mice were inoculated with MDA-MB-231 cells and received i.p. administration of increasing doses of GA-Me (4, 8, 16 and 32 mg/kg) three times weekly for eight weeks. Results showed that the highest dose of GA-Me significantly reduced tumor volume. Furthermore, tumor tissue samples were examined for angiogenesis and apoptosis by immunohistochemistry, staining with CD34 antibody, an angiogenesis marker and TUNEL analyses, respectively. Data demonstrated a gradual reduction in the number of CD34-positive vessels and an increase in the apoptosis index in the tumors of GA-Me-treated mice when compared to that of tumors in the controls [36]. Since FFLZ inhibits BC cell migration and alters the EMT phenotype, Tsao et al. investigated the antitumor activity of FFLZ. Using 4T1-bearing BALB/c mice model, they demonstrated that FFLZ reduces tumor weight and tumor volume [51]. All of these findings provide evidence of the potential of *G. lucidum* as an anti-BC therapy and provide other researchers with tools to further investigate the antitumor properties of other *Ganoderma* species.

### 6.2. Chemoprotective and Chemopreventive Effects

#### 6.2.1. Animal Models

The potential of *Ganoderma* spp. as a preventive agent against the adverse effects of chemotherapy has been evaluated in recent years. A study was done to evaluate the protective property of a water-soluble extract from the culture medium of *G. lucidum* mycelia. Researchers administered various doses of the extract to B6C3F1/Crlj mice one week before treatment with 5-fluorouracil (5-FU), tegafur with uracil (UFT), cisplatin, cyclophosphamide or gefitinib, and then, they evaluated damages to the small intestine. *G. lucidum* protected against 5-FU-induced small intestinal injury and attenuated the extent of UFT or cisplatin-induced small intestinal injury. Moreover, cyclophosphamide or gefitinib plus *G. lucidum* mycelia extract promoted crypt regeneration [123]. Additionally, the polysaccharide (PSG-1) from *G. atrum* was used to study its chemoprotective effects in cyclophosphamide-treated mice. This study revealed that PSG-1 treatment accelerated recovery dose-dependently of hemopoietic function, serum cytokines and lymphocyte activity and also significantly increased the total antioxidant capacity [124]. Another chemotherapeutic agent used to treat a broad type of malignancies, including some forms of BC, is the platinum agent cisplatin. However, long-term cisplatin use may cause nephrotoxicity [125,126]. Interestingly, an in vivo study showed that oral administration of *G. lucidum* fruiting body terpenes prevents increases in urea and creatinine levels in cisplatin treated mice. Moreover, decreases in alkaline phosphatase (ALP) activity and enhanced renal antioxidant defense were also observed [127]. Additionally, *G. lucidum* has been evaluated to assist with common secondary effects of chemotherapy and radiation. An extract of *G. lucidum* attenuated cisplatin-induced nausea and vomiting and significantly increased the food intake of rats that was originally decreased after cisplatin treatment [128]. The effectiveness of DNA vaccines has been demonstrated in several animal models and is a promising therapy against several human diseases, including cancer. Although there are advantages to DNA immunization, a reduced level of immunogenicity has been identified as an impediment for their efficacy [129]. Thus, it is necessary to obtain an adequate adjuvant to overcome this limitation. In 2011, Lin et al. studied the potential of rLZ-8 as an adjuvant for the HER2 DNA vaccine against p185^neu^ in MBT-2 cells in a mouse model of murine bladder carcinoma. The results showed that rLZ-8 increased the antitumor activity of the vaccine, promoting its Th1 response via the activation of DCs, evidencing the adjuvant potential of rLZ-8 [130]. Based on this evidence, the authors anticipate that further clinical trials administering anticancer DNA vaccines along with rLZ-8 as an adjuvant agent are possible [131].

#### 6.2.2. BC Patients

Because of its promising anticancer properties, *Ganoderma* spp. is gaining popularity among cancer patients who use it as alternative medicine. Researchers from Australia and China evaluated the clinical effects of *G. lucidum* on long-term survival, tumor response, host immune functions, adverse effects and QOL of cancer patients. The results show that patients who take *G. lucidum* alongside chemo- or radio-therapy were more likely to respond positively versus chemo- or radio-therapy alone. *G. lucidum* treatment alone did not demonstrate the same regression rate as that seen in combined therapy [132]. Another physical impairment that normally occurs during cancer therapy is a vulnerable immune system. A study of 105 cancer patients receiving chemotherapy or radiotherapy treated with a mixture of citronellol and extracts of *G. lucidum* and Chinese medicinal herbs showed an improvement in the patients’ immune function [133]. However, in a study conducted with BC patients who received chemotherapy or radiotherapy and were treated with a mixture of Chinese medicinal herbs and *G. tsugae*, improvements in the immune system markers were not statistically significant when compared with patients in the control group [134]. Therefore, the *Ganoderma* spp. used may influence therapy response.

## 7. *Ganoderma* spp. and BC Patient Behavioral Comorbidities

BC survivors who have gone through radiation, chemotherapy or surgery develop symptoms that affect not only their QOL, but also their OS [135,136,137]. These conditions include behavioral comorbidities, such as fatigue, pain, anxiety and depression. Cancer-related fatigue is a stressful and persistent sense of physical, emotional and cognitive tiredness related to cancer or cancer treatment and is not proportional to physical activity [138]. Studies show that fatigue in BC patients undergoing hormone therapy could be a result of endocrine therapy or BC treatment-induced amenorrhea (premature menopause) attributable to ovarian toxicity caused by chemo- or radio-therapy and adjuvant endocrine treatment side effects [139,140,141]. Other factors include anemia, heart disease, metabolic abnormalities and emotional symptoms (anxiety and depression) [142,143]. A study details the effects of *Ganoderma* spp. on behavioral comorbidities in BC patients. A total of 48 BC patients with CRF undergoing hormone therapy were randomized into the experimental or control groups. The experimental group was administered 1000 mg of *G. lucidum* spore powder three times a day for four weeks, while the control group received placebo for four weeks. Patients were administered the Functional Assessment of Cancer Therapy–Fatigue (FACT-F) and QLQ-30 QOL questionnaires and the Hospital Anxiety and Depression Scale (HANDS). The patients in the experimental group showed statistically-significant improvements in physical well-being and fatigue after the intervention. They also reported reduced anxiety and depression and improved QOL. Therefore, the results of this study suggest that *G. lucidum* spore powder may have beneficial effects on cancer-related behavioral comorbidities in BC patients undergoing hormone therapy without significant adverse effects [144]. In a large population-based BC cohort study in Shanghai, researchers evaluated the associations of the regular use of ginseng and *G. lucidum* as complementary therapy with the QOL of BC survivors during the first 36 months after diagnosis. Of the 4149 participants, 58.8% and 36.2% reported that they used *G. lucidum* at the six- and 36-month surveys, respectively. Survivors using *G. lucidum* after their BC diagnosis reported a higher social well-being score, but a lower physical well-being score, versus nonusers [145].

## 8. Conclusions

The need for a definitive cure for BC has led investigators to search for innovative ideas to eradicate this disease using natural alternatives with minimal side effects. *Ganoderma* spp. and their bioactive compounds represent a viable alternative to combat BC either alone or in combination with conventional therapies. This review summarized areas of research performed on *Ganoderma* spp. and BC models. There is evidence that the various compounds found within *Ganoderma* spp. have an inhibitory anticancer effect manifested by reduced tumor growth, induced apoptosis, cell cycle arrest, inhibitory activity against invasive behavior, gene expression modulation, DNA damage protection and concomitant inhibition of various signaling pathways. The multiple studies discussed in this review demonstrate the relevant therapeutic implications of *Ganoderma* spp. in various subtypes of BC. The bioactive compounds most studied are polysaccharides, triterpenes and immunomodulatory proteins, such as LZ-8, as well as fruiting-body or cracked-spore extracts. In the case of polysaccharides, their effects have been mainly described as immunomodulatory agents, decreasing the immunosuppression of conventional therapy. However, the complete mechanism of action of *Ganoderma* spp. is not entirely understood and deserves further study. It is known that in BC, *Ganoderma* spp. modulate ERK1/2, PI3-K, AKT and mTOR pathways, which in turn modulate AP-1, NF-kB, MMPs, IL-8 and uPA in cell and animal models. The chemosensitizing effects of *Ganoderma* spp. is a new field of study. Combining conventional therapies with alternative approaches may be an alternative to reduce tumor growth and increase OS and shows promising results for BC patients. *Ganoderma* spp. effects occur via potentiating conventional therapy actions or by reducing the adverse effects caused by conventional therapy. We also describe the use of *Ganoderma* spp. on fatigue and QOL in BC patients. Results from these studies could provide evidence for efficacy and thus may be used to design more comprehensive studies in the future. Thus, *Ganoderma* spp. may be used as an effective, complementary anticancer approach for chemoprevention and as an adjuvant treatment for BC. Future research includes further detailed characterization of *Ganoderma* spp. compounds, as well as clinical trials to further assess their clinical efficacy and chemopreventive effect in BC patients and survivors.

## Figures and Tables

**Table 1:** Cytostatic effects of *Ganoderma* spp.

Source	BC Cell Line	Effect	Reference
ReishiMax GLp^®^	MDA-MB-231	G0/G1 cell cycle arrest; downregulation of cyclin D1 and CDK4	[17]
ReishiMax GLp^®^	SUM-149	Downregulation of *CCND1* and *WEE*; downregulation of CCNA2, CCNB2	[23]
BreastDefend™	MDA-MB-231	Upregulation of GADD45A; downregulation of CCNDA1	[25]
*G. lucidum* ethanolic extract	MCF-7	Upregulation of p21/Waf1; downregulation of cyclin D1	[26]
*G. lucidum* ethanolic extract	MDA-MB 231	Decreased G1/S phase transition	[27]
*G. sinense* ethanolic extract	MDA-MB 231	G2 cell cycle arrest	[27]
*G. tsugae* methanolic extract	SKBR-3	G1 cell cycle arrest; downregulation of cyclins D1 and E	[28]
Ganodermanontriol	MDA-MB 231	Downregulation of CDC20	[32]
ethanol-soluble and acidic component from *G. lucidum*	MCF-7 MDA-MB-231	G1 cell cycle arrest	[34]
GADM from *G. lucidum*	MCF-7 MDA-MB-231	G1 cell cycle arrest; downregulation of total and p-Rb	[35]
GA-Me from *G. lucidum*	MDA-MB-231	Downregulation of cyclin D1	[36]
FIP-gat from *G. atrum*	MDA-MB-231	G1 cell cycle arrest	[55]

**Table 2:** Anti-breast-cancer mechanisms of *Ganoderma* spp. FIP, fungal immunomodulatory protein; ESAC, ethanol-soluble and acidic component.

Compound	Target Molecule	Effect	Biological Function	References
G. tsugae; ReishiMax GLp^®^	AKT, p-AKT	Downregulated	Cell survival, proliferation	[17,21,23,28]
ReishiMax GLp^®^	AP-1	Downregulated	Proliferation, migration	[59,61,69]
*G. lucidum*	Bax	Upregulated	Apoptosis	[26]
GA-Me	BCL-2	Downregulated	Survival	[36]
*G. neo-japonicum*; SeGLP-2B-1	Caspase 3	Upregulated	Apoptosis	[24,40]
*G. lucidum*; Khz	Caspase 7	Upregulated	Apoptosis	[26,53]
SeGLP-2B-1; Khz	Caspase 8	Upregulated	Apoptosis	[40,53]
SeGLP-2B-1; Khz	Caspase 9	Upregulated	Apoptosis	[40,53]
Ganodermanontriol	CDC20	Downregulated	Cell cycle	[32]
GAEE	CDC42	Downregulated	Migration	[37]
ReishiMax GLp^®^	CDK4	Upregulated	Cell cycle	[17]
GA-Me; ReishiMax GLp^®^	c-Myc	Downregulated	Cell survival, proliferation, oncogenesis	[19,23,31,36]
BreastDefend™	CXCR4	Downregulated	Inflammation, metastasis	[70]
BreastDefend™	Cyclin A1	Downregulated	Cell cycle	[25]
ReishiMax GLp^®^	*Cyclin A2*	Downregulated	Cell cycle	[23]
ReishiMax GLp^®^	*Cyclin B2*	Downregulated	Cell cycle	[23]
ReishiMax GLp^®^; *G. lucidum* *G. tsugae*; GA-Me	Cyclin D1	Downregulated	Cell cycle	[17,23,26,28,31]
*G. tsugae*	Cyclin E	Downregulated	Cell cycle	[28]
FIP-gat	*DUSP1*	Downregulated	Proliferation	[55]
ReishiMax GLp^®^	E-cadherin	Downregulated	Migration, invasion	[23]
ReishiMax GLp^®^	EGFR	Downregulated	Cell survival, proliferation	[21]
ReishiMax GLp^®^	*EIF4B*	Downregulated	Protein synthesis	[23]
ReishiMax GLp^®^	eIF4G	Downregulated	Protein synthesis	[23]
ReishiMax GLp^®^	ERK2, p-ERK1/2	Downregulated	Cell survival, proliferation	[21,23]
ReishiMax GLp^®^	ERα	Downregulated	Oncogenesis	[19]
GAEE	FAK	Downregulated	Migration	[37]
ReishiMax GLp^®^	*FOS*	Upregulated	Proliferation	[23]
BreastDefend™	*GADD45A*	Upregulated	Cell cycle	[25]
ReishiMax GLp^®^	*GJA1*	Downregulated	Cell signaling	[23]
*G. tsugae*	HER2, p-HER2	Downregulated	Cell survival, proliferation	[28]
GA-Me	IL-8	Downregulated	Migration, invasion	[36]
GA-Me	IL-6	Downregulated	Migration, invasion	[36]
ReishiMax GLp^®^	*JUN*	Upregulated	Proliferation	[23]
ReishiMax GLp^®^; GA-Me	MMP-2	Downregulated	Invasion, metastasis	[20,36]
ReishiMax GLp^®^; GA-Me	MMP-9	Downregulated	Invasion, metastasis	[20,36]
ReishiMax GLp^®^	*NFKBIA*	Upregulated	Proliferation, invasion	[23]
ReishiMax GLp^®^; GA-Me; BreastDefend™	NF-κB	Downregulated	Proliferation, invasion	[19,36,58,59,61,69,70]
ReishiMax GLp^®^	p120-catenin	Downregulated	Cell survival, proliferation	[23]
*G. lucidum*	p21/Waf1	Upregulated	Apoptosis	[26]
ReishiMax GLp^®^	p-4E-BP1	Downregulated	Protein synthesis	[23]
ReishiMax GLp^®^	p70S6K	Downregulated	Protein synthesis	[23]
ReishiMax GLp^®^	PAK1	Downregulated	Proliferation, migration	[23]
*G. lucidum*; ESAC; SeGLP-2B-1	PARP	Cleaved	Apoptosis	[26,34,40]
GAEE	paxillin	Downregulated	Migration	[37]
ReishiMax GLp^®^	PDK1	Downregulated	Cell survival, proliferation	[23]
ReishiMax GLp^®^	mTOR, p-mTOR	Downregulated	Cell survival, proliferation	[23]
GAEE	Rac1	Downregulated	Migration	[37]
ReishiMax GLp^®^	RAS	Downregulated	Cell survival, proliferation	[23]
GADM	Rb, p-Rb	Downregulated	Cell cycle	[35]
GAEE	RhoA	Downregulated	Migration	[37]
ReishiMax GLp^®^	S6, p-S6	Downregulated	Protein synthesis	[23]
FFLZ	TGFRβ/Smad2/3-Smad4-Snail/Slug-axis	Downregulated	EMT, metastasis	[51]
FIP-gat	*SQSTM1*	Downregulated	Autophagy, apoptosis	[55]
FIP-gat	*TNFSF8*	Downregulated	Proliferation	[55]
ganoderic acid; ReishiMax GLp^®^; BreastDefend™	uPA/uPAR	Downregulated	Migration, invasion, metastasis	[59,61,69,70]
GA-Me	VEGF	Downregulated	Angiogenesis	[36]
*G. lucidum*	*WEE*	Downregulated	Cell cycle	[23]
